# Smooth ROC curve estimation via Bernstein polynomials

**DOI:** 10.1371/journal.pone.0251959

**Published:** 2021-05-25

**Authors:** Dongliang Wang, Xueya Cai

**Affiliations:** 1 Department of Public Health and Preventive Medicine, State University of New York Upstate Medical University, Syracuse, New York, United States of America; 2 Department of Biostatistics and Computational Biology, University of Rochester, Rochester, New York, United States of America; Roswell Park Cancer Institute, UNITED STATES

## Abstract

The receiver operating characteristic (ROC) curve is commonly used to evaluate the accuracy of a diagnostic test for classifying observations into two groups. We propose two novel tuning parameters for estimating the ROC curve via Bernstein polynomial smoothing of the empirical ROC curve. The new estimator is very easy to implement with the naturally selected tuning parameter, as illustrated by analyzing both real and simulated data sets. Empirical performance is investigated through extensive simulation studies with a variety of scenarios where the two groups are both from a single family of distributions (symmetric or right skewed) or one from a symmetric and the other from a right skewed distribution. The new estimator is uniformly more efficient than the empirical ROC estimator, and very competitive to eleven other existing smooth ROC estimators in terms of mean integrated square errors.

## Introduction

The Receiver Operating Characteristic (ROC) curve has been widely used in medical research to evaluate the classification accuracy of a diagnostic test or biomarker with a continuous scale in disease screening and diagnosis. The ROC curve is essentially a plot of sensitivity versus 1-specificity, both of which are associated with the binary test rule derived at each possible threshold point. Let *X*_1_ and *X*_0_ denote diagnostic biomarker values from the diseased (case) and non-diseased (control) populations with distribution functions *X*_1_ ∼ *F*_1_ and *X*_0_ ∼ *F*_0_, respectively. The ROC curve is formally defined as $R\left(u\right)=1-F_{1}\left(F_{0}^{-1}\left(1-u\right)\right)$ for 0 ≤ *u* ≤ 1, or equivalently, as the graph (1 − *F*_0_(*x*), 1 − *F*_1_(*x*)) for all possible cutoff points *x*, where 1 − *F*_1_(*x*) and *F*_0_(*x*) are the sensitivity and specificity given a cutoff point *x*, respectively. More details about ROC analysis can be found in the books by Pepe [1] and Zhou *et al*. [2].

An extensive array of estimators for ROC curve has been developed from perspectives of parametric, nonparametric, semiparametric and Bayesian statistics. The most widely used parametric ROC estimator is the binormal model in combination with Box-Cox transformation, as described by Hanley [3], but many parametric alternatives, such as the bi-gamma model, are available as well. As an instance of semiparametric estimators, Cai & Moskowitz [4] derived maximum likelihood methods directly for a binormal ROC curve with respects to the intercept and slope parameters, instead of the distributions of test results. Empirical ROC (eROC) curve is a widely used nonparametric estimator based upon empirical distribution functions ${\hat{F}}_{i}^{},i=0,1$. The properties of the eROC have been thoroughly studied by Hsieh and Turnbull [5]. To overcome the lack of smoothness of the eROC estimator, a variety of smoothed estimators have been developed, which can be roughly distinguished into two classes. One class of the smoothed ROC estimators is based upon the classic kernel density estimators with regards to *F*_0_ and *F*_1_, and includes those proposed by Sheather and Jones [6], Altman and Leger [7], Lloyd [8], and Hall and Hyndman [9]. A comprehensive simulation study conducted by Zhou and Harezlak [10] suggests that the ROC estimator developed by Altman & Leger [7] generally performs better within this class. More recently, Qiu & Le [11] proposed a plug-in estimator replacing $F_{0}^{-1}$ with the quantile function estimator by Harrell and Davis [12]. Rufibach [13] derived a new estimator based upon the log-concave density estimates. The other class of smoothed estimators are obtained by directly smoothing the eROC, such as with local polynomial [14], or with splines [15, 16]. A more recent estimator proposed by Pulit [17] is defined as the kernel density estimator of the derived data $\left(1-{\hat{F}}_{0}^{}\left(X_{1:j}^{}\right)\right),\;j=1,\ldots ,n_{1}$, where *X*_1:*j*_ is the *j*th order statistic of the *X*_1_ values. It is worthy noting that the ROC curve estimators mentioned in this paper are only a subset of the available estimators and the readers are referred to Gonçalves *et al*. [18] for a comprehensive review, in particular for the discussions of related Bayesian methods.

More recently, Wang *et al*. [19] proposed a nonparametric ROC estimator via smoothing the Bernstein polynomials (BP). The asymptotic properties of the estimator have been well studied but more efforts are desired to select the optimal tuning parameter for real data analysis. In this study a novel Bernstein polynomial ROC estimator is derived from the framework of smooth quantile function estimation. The new estimator shares the asymptotic properties with the existing Bernstein polynomial estimator but naturally provides an inherent bandwidth. The empirical performances of the new estimators are investigated and compared with a wide range of existing ROC estimators via extensive simulation studies.

We should start from a formal introduction of Bernstein polynomial approximation. A continuous function *f*(*x*) on the interval [0, 1] can be approximated by

$$\begin{array}{c} B_{n}^{}\left(f\left(x\right)\right)=\sum_{k=0}^{n}w_{k,n}^{}\left(x\right)f\left(k/n\right), \end{array}$$

where $w_{k,n}^{}\left(x\right)=\left(\begin{array}{c} n\\ k \end{array}\right)x_{}^{k}{\left(1-x\right)}_{}^{n-k}$, and as *n* → ∞,

$$\begin{array}{c} B_{n}^{}\left(f\left(x\right)\right)\to f\left(x\right). \end{array}$$

Bernstein polynomials have been previously used for estimating probability density function [20], cumulative distribution function [21], and quantile function [22].

The rest of the paper is organized as follows: In the next section, a new estimator of the ROC curve is proposed via directly smoothing the eROC curve with Bernstein polynomial approximation. The BP method is illustrated by analyzing both simulated and real data sets, respectively. Empirical performance of the BP estimators is further explored via extensive simulations.

## Bernstein polynomial ROC estimator

We start this section by formally defining the empirical ROC estimator. Let $X_{1}=\left\{X_{11},X_{12},\ldots ,X_{1n_{1}^{}}\right\}$ and $X_{0}=\left\{X_{01},X_{02},\ldots ,X_{0n_{0}^{}}\right\}$ denote *n*_1_ and *n*_0_ independent diagnostic biomarker measurements from the diseased (case) and non-diseased (control) populations with distribution functions *X*_1_ ∼ *F*_1_ and *X*_0_ ∼ *F*_0_, respectively. Let *X*_*i*:*j*_ (*i* = 1, 0, *j* = 1, …, *n*_*i*_), denote the *j*th order statistic from the *i*th population. Given **X**_**i**_, the empirical distribution functions and the correspondent sample quantile functions are defined as

$$\begin{array}{ccc} {\hat{F}}_{i}^{}\left(x\right) & = & \frac{1}{n_{i}^{}}\sum_{j=1}^{n_{i}}\text{I}\left(X_{ij}^{}\leq x\right), \end{array}$$ (0.1)

$$\begin{array}{ccc} {\hat{F}}_{i}^{-1}\left(u\right) & = & X_{i:\lfloor n_{i}u\rfloor +1}^{}, \end{array}$$ (0.2)

respectively, where *I*(⋅) is the indicator function and ⌊⋅⌋ is the floor function. The ‘+1’ term in ${\hat{F}}_{i}^{-1}\left(u\right)$ ensures that sample quantiles lie between the minimum and maximum order statistics. The empirical ROC curve estimator is defined as

$$\begin{array}{c} \hat{R}\left(u\right)=1-{\hat{F}}_{1}\left({\hat{F}}_{0}^{-1}\left(1-u\right)\right),\;\text{over}\;u\in \left[0,1\right]. \end{array}$$ (0.3)

The empirical ROC curve is essentially a step function of the proportions $\left(1-{\hat{F}}_{1}\left(x\right)\right)$ versus $\left(1-{\hat{F}}_{0}\left(x\right)\right)$ where the steps take place at every possible values of **X**_**1**_.

**Definition 0.1**. *The Bernstein polynomial ROC (bpROC) estimator is defined as*

$$\begin{array}{c} B_{n_{0}}\left(R\right)\left(u\right)=\tilde{R}\left(u\right)=\sum_{j=0}^{n_{0}}w_{j,n_{0}}\left(u\right)(1-{\hat{F}}_{1}\left(X_{0:j}^{}\right)), \end{array}$$ (0.4)

*where* *X*_0:0_ = −∞.

The definition of the estimator at (0.4) follows similarly to the derivation of the Bernstein polynomial quantile function estimator [22]. Given a continuous distribution function *F*_0_,

$$\begin{array}{c} P(F_{0}^{-1}\left(1-u\right)\in \left[X_{0:k}^{},X_{0:k+1}^{}\right))=\left(\begin{array}{c} n_{0}\\ k \end{array}\right){\left(1-u\right)}_{}^{k}u_{}^{n_{0}^{}-k}. \end{array}$$

It follows directly that

$$\begin{array}{ccc} & & P(R\left(u\right)\in [1-F_{1}\left(X_{0:k}^{}\right),1-F_{1}\left(X_{0:k+1}^{}\right))) \end{array}$$ (0.5)

$$\begin{array}{ccc} & = & P(1-F_{1}\left(F_{0}^{-1}\left(1-u\right)\right)\in [1-F_{1}\left(X_{0:k}^{}\right),1-F_{1}\left(X_{0:k+1}^{}\right))) \end{array}$$

$$\begin{array}{ccc} & = & \left(\begin{array}{c} n_{0}\\ k \end{array}\right){\left(1-u\right)}_{}^{k}u_{}^{n_{0}^{}-k}. \end{array}$$

Thus a class of L-estimators for *R*(*u*) can be constructed as

$$\begin{array}{c} \hat{R}\left(u\right)=\sum_{j=0}^{n_{0}}w_{j,n_{0}}\left(u\right)h(1-{\hat{F}}_{1}\left(X_{0:k}^{}\right),1-{\hat{F}}_{1}\left(X_{0:k+1}^{}\right)),\;\text{for}\;u\in \left[0,1\right]. \end{array}$$

Letting $h(1-F_{1}\left(X_{0:k}^{}\right),1-F_{1}\left(X_{0:k+1}^{}\right))=1-{\hat{F}}_{1}\left(X_{0:k}^{}\right)$ leads to the Bernstein polynomial ROC estimator at (0.4). Note that the function *h*(*s*, *t*) can be any measurable function such that *h*(*s*, *t*) ∈ [*s*, *t*).

The bpROC estimator at (0.4) is a special case of the estimators recently proposed by Wang *et al*. (2019), namely,

$$\begin{array}{c} B_{m}\left(\hat{R}\left(u\right)\right)=\tilde{R}\left(u\right)=\sum_{j=0}^{m}w_{j,m}\left(u\right)\hat{R}\left(j/m\right), \end{array}$$

where

$$\begin{array}{c} w_{j,m}\left(u\right)=\left(\begin{array}{c} m\\ j \end{array}\right)u_{}^{j}{\left(1-u\right)}_{}^{m-j}. \end{array}$$

Wang *et al*. [19] have thoroughly investigated the asymptotic properties but also noted that “the choice of the tuning parameter is generally an issue” and “are going to put more effort into the tuning method of parameter *m*”. The contribution of the bpROC estimator at (0.4) is to provide a naturally selected bandwidth *m* = *n*_0_, which reflects the numbers of steps in ${\hat{F}}_{0}^{}$. Since the ROC estimation depends on both *F*_0_ and *F*_1_, and there are some equal values among $1-{\hat{F}}_{1}\left(X_{0:j}^{}\right),\;j=0,\ldots ,n_{0}$, we take one step further and propose another bandwidth *m* as the number of steps of the empirical ROC curve including the two end points (0, 0) and (1, 1), in case that one or both of these two points are not included in the eROC curve.

Both bpROC estimators avoid burden of bandwidth selection by automatically tuning the bandwidth along with sample sizes, and yield satisfactory empirical performance as shown in Section. The new BP ROC estimators are also transformation invariant, since they are direct derivatives of eROC based on rank statistics.

## Examples

**Fig 1:**
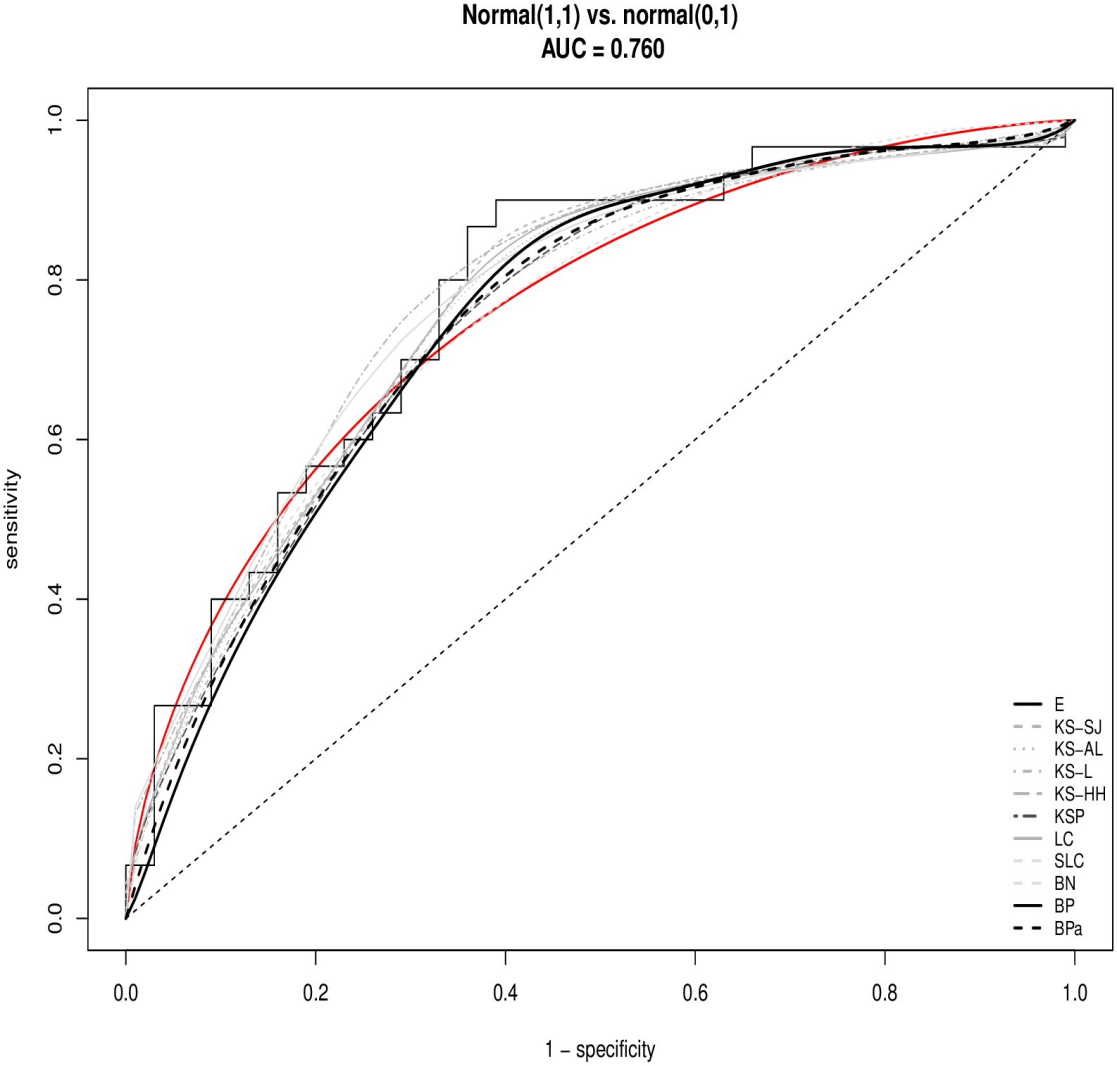
The estimated ROC curves from a simulated example data set. The red line is the true ROC curve.

To illustrate the bpROC estimators and compare with other existing estimators, a simulated example is firstly analyzed for the sake that the true ROC curve can be used for reference. In this example, *n* = 30 observations are generated from *F*_0_ ∼ Normal(0,1) and *F*_1_ ∼ Normal(1,1), respectively. The data are displayed in Table 1. A variety of ROC estimators are graphed in Fig 1, including the empirical ROC curve (E) and those.

- derived from kernel estimators of *F*_0_ and *F*_1_ with bandwidth selected by Sheather and Marron [6] (KS-SJ), by Altman and Leger [7] (KS-AL), by Lloyd [8] (KS-L), and by Hall and Hyndman [9] (KS-HH),
- derived by kernel smoothing of pseudo data by Pulit [17] (KSP),
- derived from log-concave density estimation (LC) and (SLC) [13],
- derived from binormal model (BN),
- derived by Bernstein Polynomials with *m* equal to the number of unique values from *F*_0_ (BP) and equal to the number of steps in empirical ROC estimate (BPa).

**Table 1:** A simulated data set with *n* = 30 from *F*_0_ ∼ Normal(0, 1) and *F*_0_ ∼ Normal(1, 1), respectively.

Cases	-1.661, 0.945, 0.495, 0.473, 1.498, 1.011, 0.682, 1.943, -0.543, 0.188,0.835, 2.976, 1.465, 2.056, 3.178, 0.619, 2.09, 0.551, 2.296, 2.373, 0.637, 1.196, -0.049, 0.169, 0.809, 0.735, 1.844, 1.446, 1.093, -0.531
Controls	-0.417, -0.763, -0.9, -0.65, -0.396, -0.737, 0.827, -0.406, 0.614, 0.065, -1.46, 1.018, -0.824, 0.755, -0.539, -0.819, 0.399, -0.384, -0.203, 1.148, 0.658, 0.7, -0.632, 1.569, -0.72, 2.378, -0.49, -1.16, 1.779, -0.152

A brief description of each method has been provided in the introduction. A variety of R packages can be used to calculate the existing ROC estimators. In this paper, the R package *pROC* is used to calculate the KS-SJ, KS-AL, and BN estimators. The R code available from https://rdrr.io/bioc/ROC/src/R/ROC.hyndman.R is used for KS-L and KS-HH estimators. The R package *logcondens* is used for calculating LC and SLC estimators. All smoothed ROC estimators provide a reasonable amount of smoothing to the empirical ROC estimator and all estimators demonstrate acceptable accuracy for estimating the true ROC curve.

**Fig 2:**
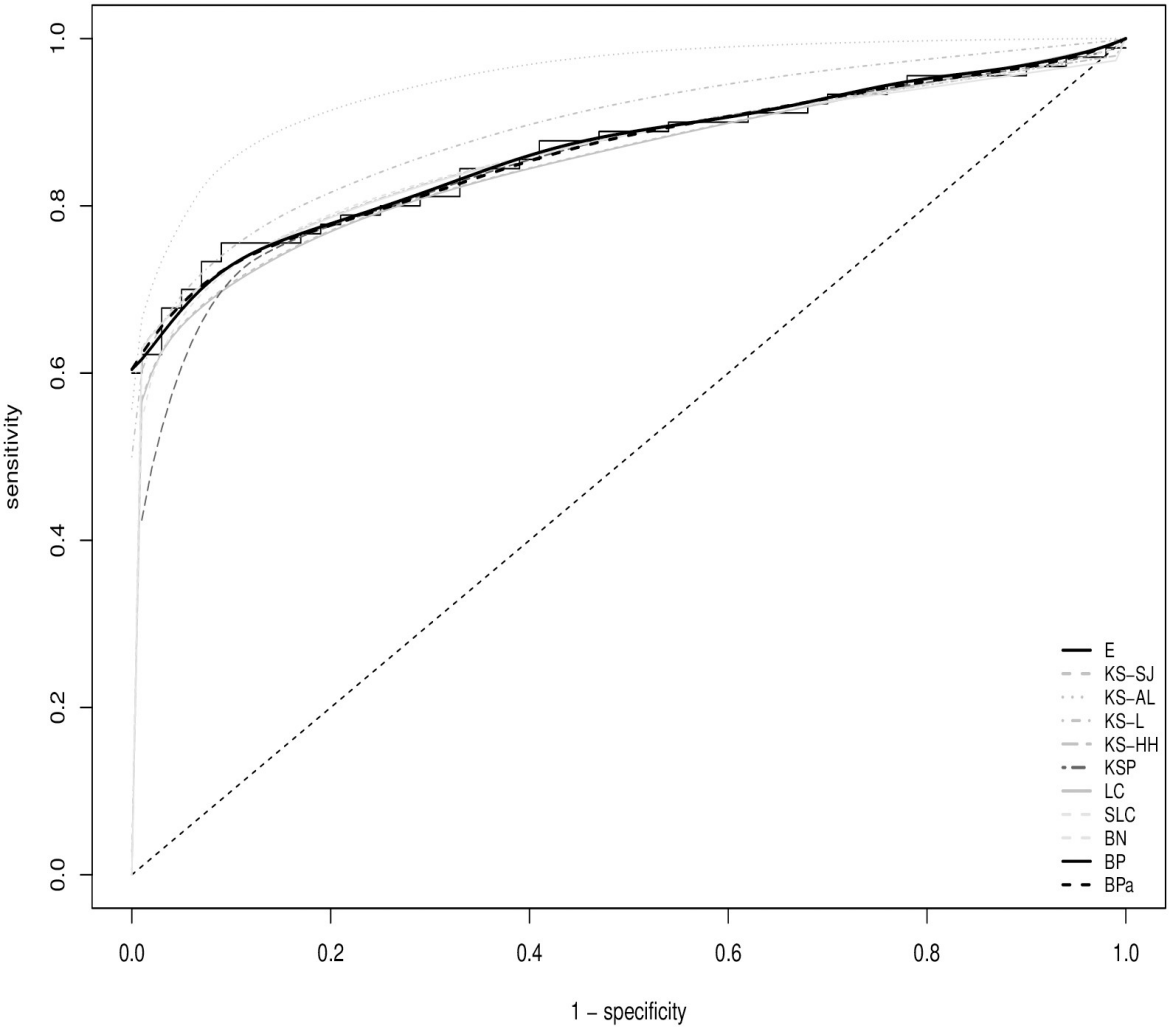
The estimated ROC curves from the pancreas data.

To further illustrate the application of the estimation methods on real life data, the well known pancreas data, firstly published by Wieand *et al*. [23], is analyzed to evaluate the capacity of a carbohydrate antigen (CA19.9) to distinguish subjects with pancreatic cancer (*n*_1_ = 90) from those with pancreatitis but not pancreatic cancer (*n*_0_ = 51). The ROC estimates from this data set are displayed in Fig 2. Other than KS-L and KS-HH, all the ROC estimates share high similarity, particularly when the specificity is less than 80%.

## Simulation study

To examine the empirical performance of bpROC estimators as compared with other existing methods, a fairly comprehensive simulation study is performed under a variety of scenarios that *F*_0_ and *F*_1_ are from either a symmetric (normal) or right skewed (gamma) distribution with the area under the ROC curve (AUC) approximately equal to 0.70 and 0.90 for the evaluation of a moderately good and excellent biomarker, respectively. More specifically, six sampling scenarios are examined as below,

- S1. *F*_1_ ∼ normal(1,1) and *F*_0_ ∼ normal(0,1) with AUC = 0.760;
- S2. *F*_1_ ∼ normal(2,1.2) and *F*_0_ ∼ normal(0,1) with AUC = 0.900;
- S3. *F*_1_ ∼ gamma(0.5,4) and *F*_0_ ∼ gamma(0.5,1) with AUC = 0.702;
- S4. *F*_1_ ∼ gamma(2,0.5) and *F*_0_ ∼ gamma(1,1) with AUC = 0.887;
- S5. *F*_1_ ∼ gamma(2,2) and *F*_0_ ∼ normal(2,1) with AUC = 0.725;
- S6. *F*_1_ ∼ gamma(2,2) and *F*_0_ ∼ normal(1,1) with AUC = 0.870.

Scenarios S5 and S6 are of particular interest since that in reality it is not uncommon that the distribution becomes right-skewed from symmetric when subjects are diseased. Four different sample sizes are considered with (*n*_0_, *n*_1_) equal to (30, 30), (30, 100), (100,30), (100, 100), respectively. For each given sample size, data are generated from the desired distributions and the 11 ROC estimators as listed in Section are calculated at the grid points *u*_*i*_ = 0.01 *to* 0.99 *by* 0.01, *i* = 1, …, 99. The process is replicated *N* = 2000 times.

The performance of the *j*th (*j* = 1, …, 11) ROC estimator at each grid point *u*_*i*_ is assessed by mean squared error (MSE)

$$\begin{array}{c} {\hat{MSE}}_{j}\left(u_{i}\right)=\frac{1}{N}\sum_{k=1}^{N}{\left({\hat{ROC}}_{j}\left(u_{i}\right)-ROC\left(u_{i}\right)\right)}_{}^{2}. \end{array}$$

For better presentation, we further use empirical ROC estimator as the reference and assess the performance of the *j*th ROC estimator at each grid point by relative efficiency (RE), which is defined as

$$\begin{array}{c} RE_{j}\left(u_{i}\right)=\frac{{\hat{MSE}}_{E}\left(u_{i}\right)}{{\hat{MSE}}_{j}\left(u_{i}\right)}, \end{array}$$

where ${\hat{MSE}}_{E}\left(u_{i}\right)$ is the MSE associated with the empirical ROC estimator at *u*_*i*_. Thus a value of RE greater than 1 indicates a more efficient ROC estimator with less MSE than the empirical estimator.

To assess the overall performance of each estimator across all grid points, we use mean integrated square error (MISE), which has been previously used by Zhou and Harezlak (2002) and defined as

$$\begin{array}{c} {MISE}_{j}=\frac{1}{N}\sum_{k=1}^{N}\int {\left(\hat{\text{ROC}}\left(u\right)-ROC\left(u\right)\right)}_{}^{2}du. \end{array}$$

Again for better comparison, we calculate overall relative efficiency (ORE) as

$$\begin{array}{c} ORE\left(j\right)={\hat{MISE}}_{E}/{\hat{MISE}}_{j}, \end{array}$$ (0.6)

where ${\hat{MISE}}_{E}$ is the MISE of the empirical ROC estimator. A higher ORE value indicates smaller MISE and better performance, and an ORE value greater than 1 indicates the estimator is more efficient than the empirical ROC estimator.

**Fig 3:**
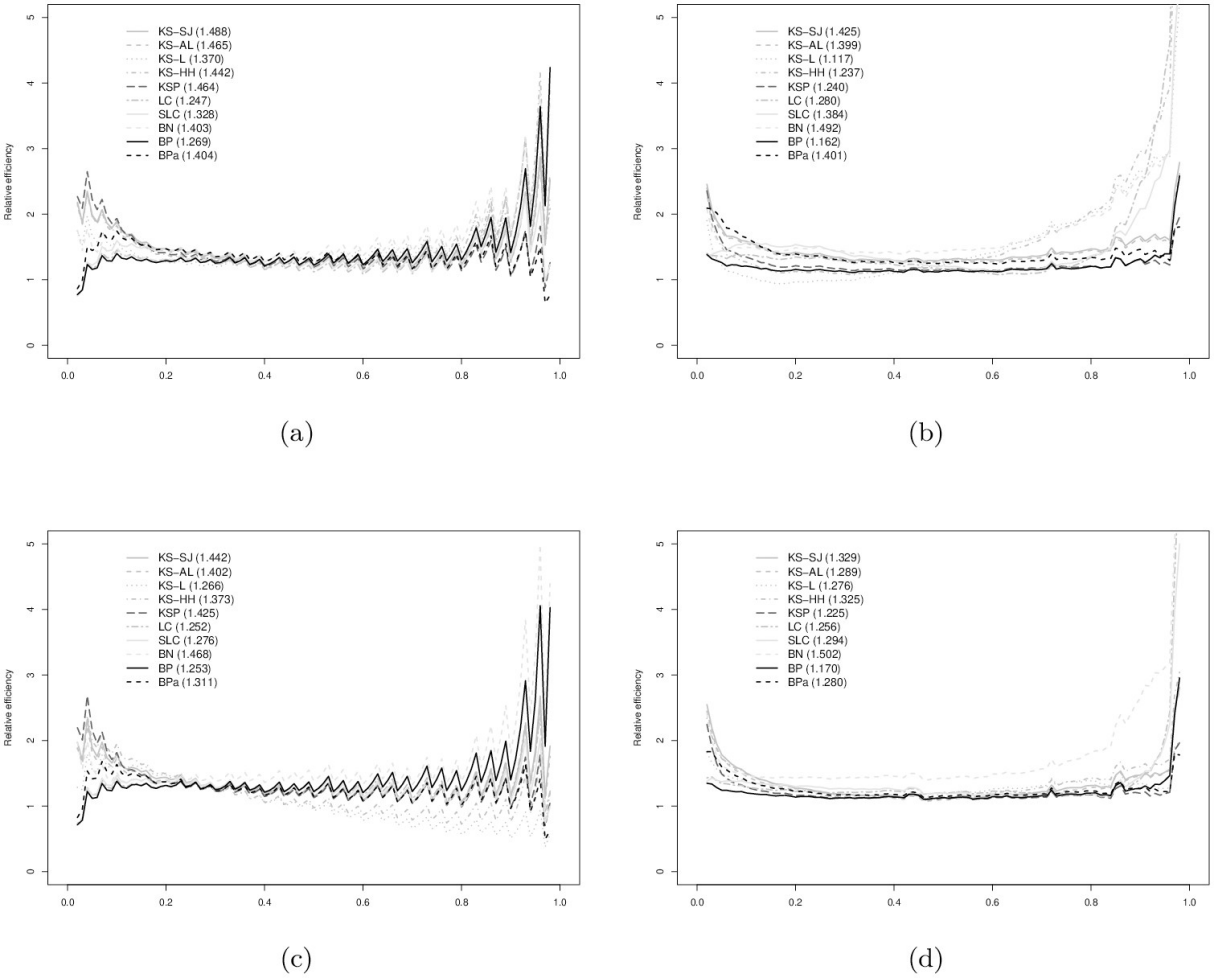
Normal(1,1) vs. normal(0,1) with AUC = 0.760: (a) *n*_1_ = 30 vs. *n*_0_ = 30; (b) *n*_1_ = 30 vs. *n*_0_ = 100; (c) *n*_1_ = 100 vs. *n*_0_ = 30; and, (d) *n*_1_ = 100 vs. *n*_0_ = 100. Overall relative efficiency at (0.1) for each smooth ROC estimator is provided in the bracket behind the abbreviation.

**Fig 4:**
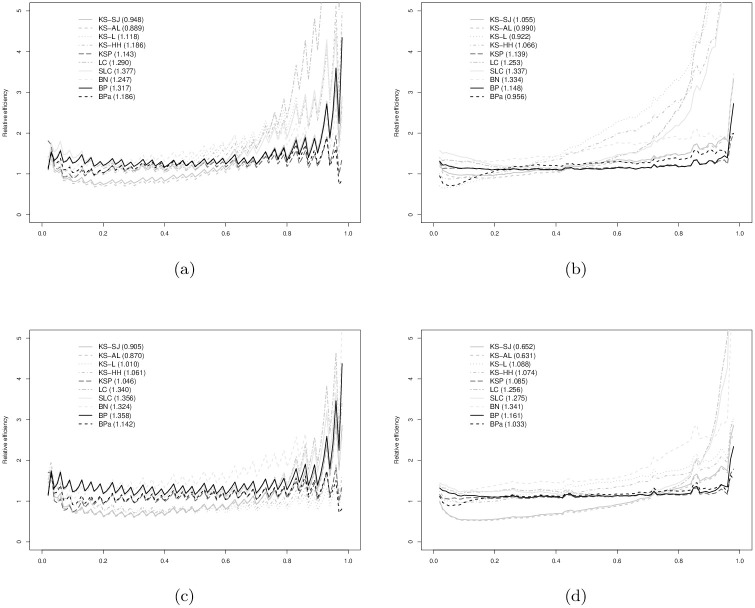
Normal(2,1.2) vs. normal(0,1) with AUC = 0.900: (a) *n*_1_ = 30 vs. *n*_0_ = 30; (b) *n*_1_ = 30 vs. *n*_0_ = 100; (c) *n*_1_ = 100 vs. *n*_0_ = 30; and, (d) *n*_1_ = 100 vs. *n*_0_ = 100. Overall relative efficiency at (0.1) for each smooth ROC estimator is provided in the bracket behind the abbreviation.

**Fig 5:**
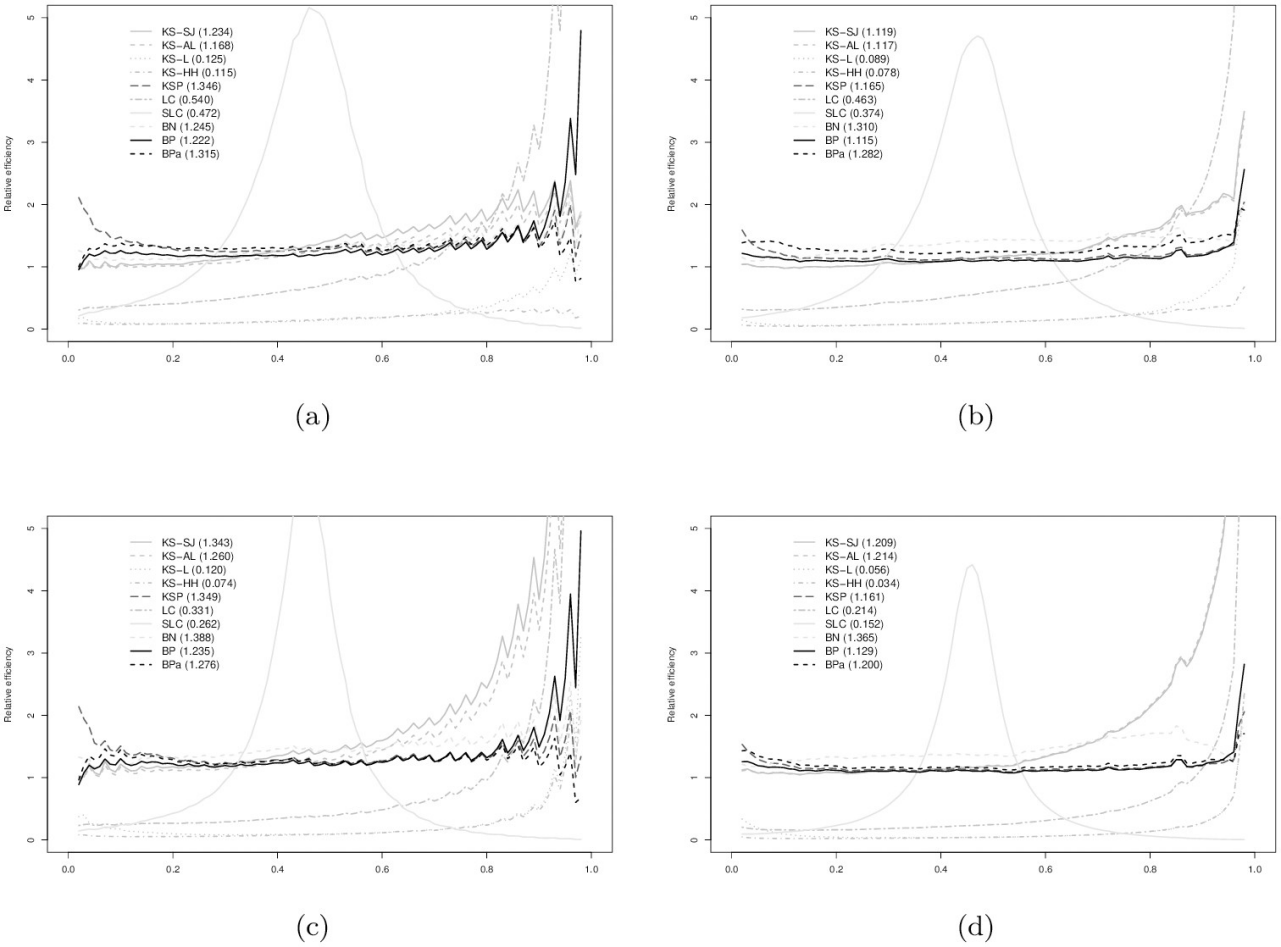
Gamma(0.5,4) vs. gamma(0.5,1) with AUC = 0.702: (a) *n*_1_ = 30 vs. *n*_0_ = 30; (b) *n*_1_ = 30 vs. *n*_0_ = 100; (c) *n*_1_ = 100 vs. *n*_0_ = 30; and, (d) *n*_1_ = 100 vs. *n*_0_ = 100. Overall relative efficiency at (0.1) for each smooth ROC estimator is provided in the bracket behind the abbreviation.

**Fig 6:**
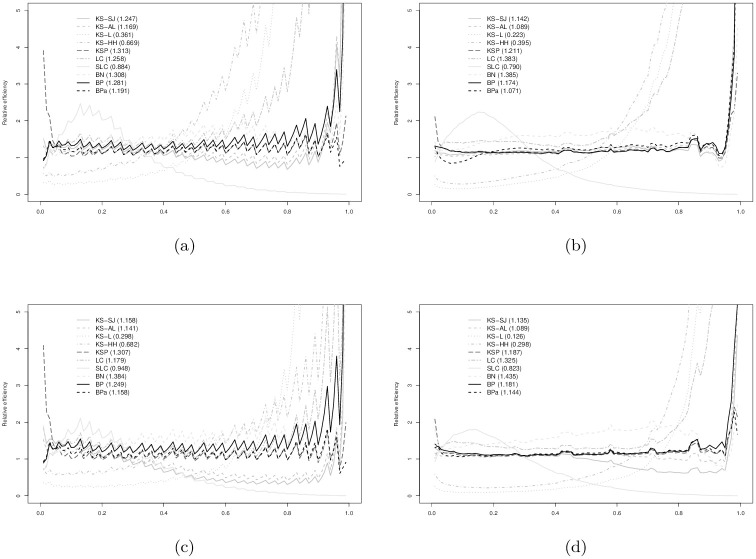
Gamma(2,0.5) vs. gamma(1,1) with AUC = 0.887: (a) *n*_1_ = 30 vs. *n*_0_ = 30; (b) *n*_1_ = 30 vs. *n*_0_ = 100; (c) *n*_1_ = 100 vs. *n*_0_ = 30; and, (d) *n*_1_ = 100 vs. *n*_0_ = 100. Overall relative efficiency at (0.1) for each smooth ROC estimator is provided in the bracket behind the abbreviation.

**Fig 7:**
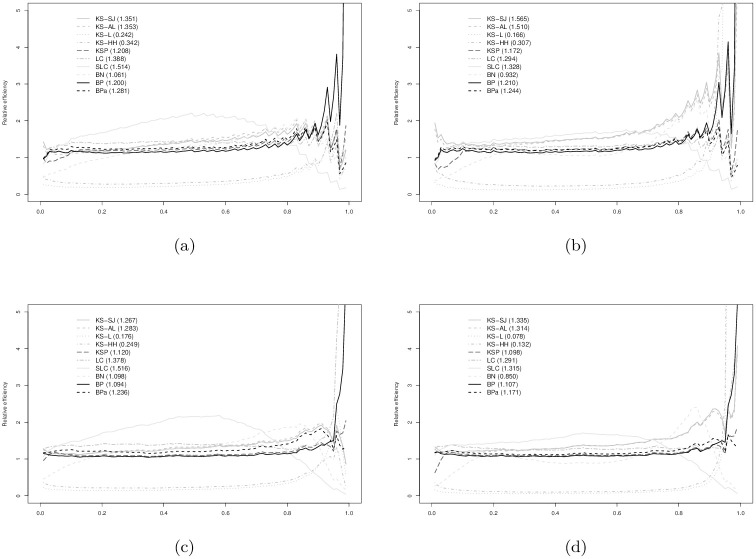
Gamma (2,2) vs. Normal(2, 1) with AUC = 0.725: (a) *n*_1_ = 30 vs. *n*_0_ = 30; (b) *n*_1_ = 30 vs. *n*_0_ = 100; (c) *n*_1_ = 100 vs. *n*_0_ = 30; and, (d) *n*_1_ = 100 vs. *n*_0_ = 100. Overall relative efficiency at (0.1) for each smooth ROC estimator is provided in the bracket behind the abbreviation.

**Fig 8:**
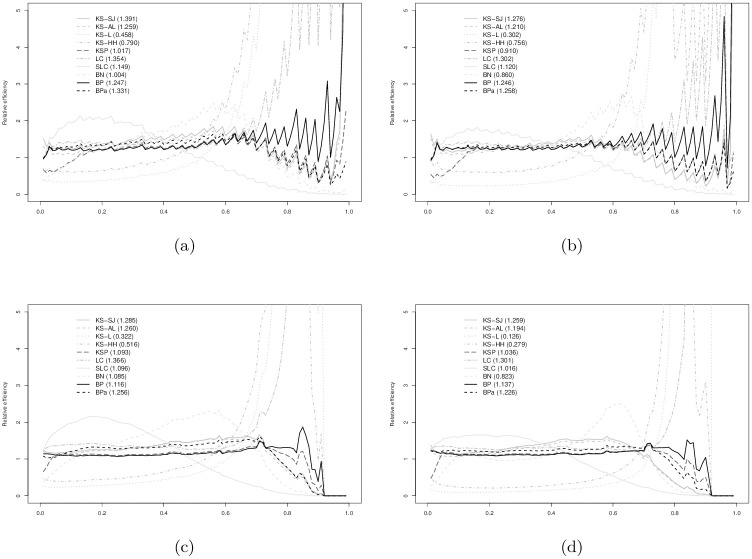
Gamma (2,2) vs. Normal(1, 1) AUC = 0.870: (a) *n*_1_ = 30 vs. *n*_0_ = 30; (b) *n*_1_ = 30 vs. *n*_0_ = 100; (c) *n*_1_ = 100 vs. *n*_0_ = 30; and, (d) *n*_1_ = 100 vs. *n*_0_ = 100. Overall relative efficiency at (0.1) for each smooth ROC estimator is provided in the bracket behind the abbreviation.

In Figs 3–8, relative efficiencies of each ROC estimator are displayed to show the pattern that the performance of each ROC estimator changes over the line (0,1), together with the overall relative efficiencies, provided at the top left corners.

Figs 3 and 4 show the results when data are simulated from normal distributions. It is not surprising that the ROC estimators from binormal model (BN) perform the best for most scenarios. For AUC = 0.760 in Fig 3, the BPa and KS-SJ are comparable to and sometimes better than the BN estimator. KSP performs reasonably well when *n*_0_ = 30 in Fig 3(a) and 3(c). For AUC = 0.900 in Fig 4, only the BP and SLC estimators are slightly worse than the BN estimator. The efficacy of BPa is slightly worse than the BP estimator.

Figs 5 and 6 show the results when data are simulated from gamma distributions. For AUC = 0.702 from Fig 5, BN demonstrates best efficiency. The performances of BP and BPa estimators are comparable to KSP but slightly worse than the BN estimator. All estimators are more efficient than the eROC estimator, other than KS-L, KS-HH, LC, and SLC. The poor performance of LC and SLC estimators is mainly caused by the violation of the log-concavity assumption. In Fig 6 with AUC = 0.883, the BN estimators still appear as the best one. The performances of LC, KSP, BP, and BPa are comparable to each other and slightly worse than the BN estimator. The efficiencies of LC and SLC estimators are significantly improved, now that the log-concavity assumption is met.

Figs 7 and 8 show the results when data are simulated from gamma and normal distributions. The relative efficacy of the BP estimators as compared to empirical ROC estimator does not change much from the binormal and bi-gamma models. BPa estimator still demonstrates as one of the most efficient estimators with comparable performance to KS-SJ, LC, and SLC estimators. The performance of the BN estimator is slightly worse than the eROC estimator in general.

As a summary of the observations from our simulation study, it is fair to conclude that the BP ROC estimators, demonstrate better and more reliable efficiency, as compared to other estimators, particularly for small sample sizes. For larger sample sizes, the performance of the BP estimator remains one of the best and is comparable to the best estimator, which usually changes given different simulation scenarios. The BPa estimator usually performs slightly better than the BP estimator.

It is also worthy noting that the BP estimators always outperform the empirical ROC estimator, when data are simulated from normal, gamma, or mixed distributions. The performance of the other estimators depends on the underlying distributions from which the data are simulated. For instance, the BN estimator does not perform well when *F*_0_ and *F*_1_ are from different families of distributions and the efficiency of the LC and SLC estimators is sensitive to the log-concavity assumption.

## Conclusion and discussion

In this paper we propose a couple of tuning parameters which can be easily implemented to the recently derived Bernstein polynomial ROC estimator. The corresponding ROC estimators in general perform better than a wide range of existing estimators in terms of MISE, under scenarios that are commonly encountered in real word data. Further studies are underway towards evaluating the proposed ROC estimator on estimating the associated summary statistics, such as the area under the ROC curve, the partial AUC, and the Youden index.

## Supporting information

S1 Datasets (DOCX)
