# Supplementary material for: Smooth ROC curve estimation via Bernstein polynomials
Source: PLoS One. 2021 May 25;16(5):e0251959. doi: 10.1371/journal.pone.0251959 (PMC8148335; doi:10.1371/journal.pone.0251959)
Supplement: S1 Datasets — (DOCX) [file pone.0251959.s001.docx]

Data set 1: A simulated data set with n=30 from Normal (0,1) and Normal (1,1), respectively.

Cases:

-1.661, 0.945, 0.495, 0.473, 1.498, 1.011, 0.682, 1.943, -0.543, 0.188,0.835,

2.976, 1.465, 2.056, 3.178, 0.619, 2.09, 0.551, 2.296, 2.373, 0.637, 1.196,

-0.049, 0.169, 0.809, 0.735, 1.844, 1.446, 1.093, -0.531

Controls:

-0.417, -0.763, -0.9, -0.65, -0.396, -0.737, 0.827, -0.406, 0.614, 0.065, -1.46,

1.018, -0.824, 0.755, -0.539, -0.819, 0.399, -0.384, -0.203, 1.148, 0.658, 0.7,

-0.632, 1.569, -0.72, 2.378, -0.49, -1.16, 1.779, -0.152

Data set 2: The pancreas data, firstly published by Wieand et al. [23].

| ca199 | status |
| --- | --- |
| 28 | 0 |
| 15.5 | 0 |
| 8.2 | 0 |
| 3.4 | 0 |
| 17.3 | 0 |
| 15.2 | 0 |
| 32.9 | 0 |
| 11.1 | 0 |
| 87.5 | 0 |
| 16.2 | 0 |
| 107.9 | 0 |
| 5.7 | 0 |
| 25.6 | 0 |
| 31.2 | 0 |
| 21.6 | 0 |
| 55.6 | 0 |
| 8.8 | 0 |
| 6.5 | 0 |
| 22.1 | 0 |
| 14.4 | 0 |
| 44.2 | 0 |
| 3.7 | 0 |
| 7.8 | 0 |
| 8.9 | 0 |
| 18 | 0 |
| 6.5 | 0 |
| 4.9 | 0 |
| 10.4 | 0 |
| 5 | 0 |
| 5.3 | 0 |
| 6.5 | 0 |
| 6.9 | 0 |
| 8.2 | 0 |
| 21.8 | 0 |
| 6.6 | 0 |
| 7.6 | 0 |
| 15.4 | 0 |
| 59.2 | 0 |
| 5.1 | 0 |
| 10 | 0 |
| 5.3 | 0 |
| 32.6 | 0 |
| 4.6 | 0 |
| 6.9 | 0 |
| 4 | 0 |
| 3.65 | 0 |
| 7.8 | 0 |
| 32.5 | 0 |
| 11.5 | 0 |
| 4 | 0 |
| 10.2 | 0 |
| 2.4 | 1 |
| 719 | 1 |
| 2106.67 | 1 |
| 24000 | 1 |
| 1715 | 1 |
| 3.6 | 1 |
| 521.5 | 1 |
| 1600 | 1 |
| 454 | 1 |
| 109.7 | 1 |
| 23.7 | 1 |
| 464 | 1 |
| 9810 | 1 |
| 255 | 1 |
| 58.7 | 1 |
| 225 | 1 |
| 90.1 | 1 |
| 50 | 1 |
| 5.6 | 1 |
| 4070 | 1 |
| 592 | 1 |
| 28.6 | 1 |
| 6160 | 1 |
| 1090 | 1 |
| 10.4 | 1 |
| 27.3 | 1 |
| 162 | 1 |
| 3560 | 1 |
| 14.7 | 1 |
| 83.3 | 1 |
| 336 | 1 |
| 55.7 | 1 |
| 1520 | 1 |
| 3.9 | 1 |
| 5.8 | 1 |
| 8.45 | 1 |
| 361 | 1 |
| 369 | 1 |
| 8230 | 1 |
| 39.3 | 1 |
| 43.5 | 1 |
| 361 | 1 |
| 12.8 | 1 |
| 18 | 1 |
| 9590 | 1 |
| 555 | 1 |
| 60.2 | 1 |
| 21.8 | 1 |
| 900 | 1 |
| 6.6 | 1 |
| 239 | 1 |
| 3100 | 1 |
| 3275 | 1 |
| 682 | 1 |
| 85.4 | 1 |
| 10290 | 1 |
| 770 | 1 |
| 247.6 | 1 |
| 12320 | 1 |
| 113.1 | 1 |
| 1079 | 1 |
| 45.6 | 1 |
| 1630 | 1 |
| 79.4 | 1 |
| 508 | 1 |
| 3190 | 1 |
| 542 | 1 |
| 1021 | 1 |
| 235 | 1 |
| 251 | 1 |
| 3160 | 1 |
| 479 | 1 |
| 222 | 1 |
| 15.7 | 1 |
| 2540 | 1 |
| 11630 | 1 |
| 1810 | 1 |
| 6.9 | 1 |
| 4.1 | 1 |
| 15.6 | 1 |
| 9820 | 1 |
| 1490 | 1 |
| 15.7 | 1 |
| 45.8 | 1 |
| 7.8 | 1 |
| 12.8 | 1 |
| 100.53 | 1 |
| 227 | 1 |
| 70.9 | 1 |
| 2500 | 1 |
